# Case Report: A PD-L1-Positive Patient With Pleomorphic Rhabdomyosarcoma Achieving an Impressive Response to Immunotherapy

**DOI:** 10.3389/fimmu.2022.815598

**Published:** 2022-03-17

**Authors:** Jiayong Liu, Peijie Liu, Fuyu Gong, Youhui Tian, Xiaochen Zhao

**Affiliations:** ^1^ Key Laboratory of Carcinogenesis and Translational Research (Ministry of Education/Beijing), Department of Bone and Soft Tissue Tumor, Peking University Cancer Hospital & Institute, Beijing, China; ^2^ Department of Oncology, The First Affiliated Hospital of Henan University, Kaifeng, China; ^3^ The Medical Department, 3D Medicines Inc., Shanghai, China

**Keywords:** pleomorphic rhabdomyosarcoma, immunotherapy, pazopanib, PD-L1, CD8 T cell

## Abstract

There is currently a lack of effective systemic treatment for patients with advanced pleomorphic rhabdomyosarcoma (PRMS). Although programmed death protein 1 (PD-1) inhibitors have shown efficacy in various solid tumors, their effects on PRMS have not been well established. Here, we present a case of a 12-year-old Chinese male adolescent with metastatic PRMS who benefited from the PD-1 inhibitor nivolumab. The patient initially underwent primary tumor resection but failed to respond to subsequent first-line chemotherapy and second-line pazopanib treatment. Pathological examination showed positive PD-L1 expression and tumor-infiltrating lymphocytes in the tumor tissue, and the patient was administered nivolumab as a posterior-line treatment. After attaining a clinically partial response (PR), surgical resection was performed, which was followed by adjuvant nivolumab. At the time of the submission of this manuscript, the patient achieved recurrence-free survival (RFS) lasting 45 months and counting. This is the first clinical evidence that a patient with refractory PRMS was controlled by anti-PD-1 antibody, with an RFS lasting more than 3 years. This case suggests that PD-L1 expression and T-cell infiltration could be used as potential biomarkers for PRMS immunotherapy.

## Introduction

Rhabdomyosarcoma (RMS), which originates from the mesenchymal tissue, is the most common type of soft tissue sarcoma (STS) that occurs in childhood and adolescence (1). According to the World Health Organization classification of soft tissue tumors, RMS can be classified into four histological subtypes: alveolar RMS (ARMS), embryonal RMS (ERMS), spindle cell/sclerosing RMS (SRMS), and pleomorphic RMS (PRMS) (2). In comparison to the other RMS subtypes, PRMS is associated with a poor response to standard chemotherapy and a poor prognosis for both local and metastatic disease (3). Nonetheless, anthracycline-based chemotherapy remains the first-line standard treatment for advanced PRMS (4, 5). Pazopanib and regorafenib are multitarget tyrosine kinase inhibitors (TKIs) that are recommended as second-line treatment options for nonspecific STS, including PRMS (6).

Monoclonal antibodies against programmed death-1 (PD-1) and programmed death-ligand 1 (PD-L1) could help activate cytolytic T lymphocytes by blocking the PD-L1/PD-1 signaling pathway to prevent tumors from achieving immune evasion. Immune checkpoint inhibitor (ICI)-based immunotherapy has shown promising efficacy in various malignancies (7–9). Evidence regarding ICI efficacy in RMS remains scarce (10). A multicenter phase 2 clinical trial showed that ICIs exhibited promising efficacy and an acceptable safety profile in advanced STS (11). However, a retrospective study revealed that RMS showed no response to the anti-PD-1 antibody pembrolizumab (12). A phase I/II study (NCT02304458) is underway to explore the use of nivolumab as a single agent or in combination with ipilimumab in refractory solid tumors, including RMS. Predictive biomarkers should be investigated to identify the subset of patients who respond to immunotherapy.

**Figure 1:**
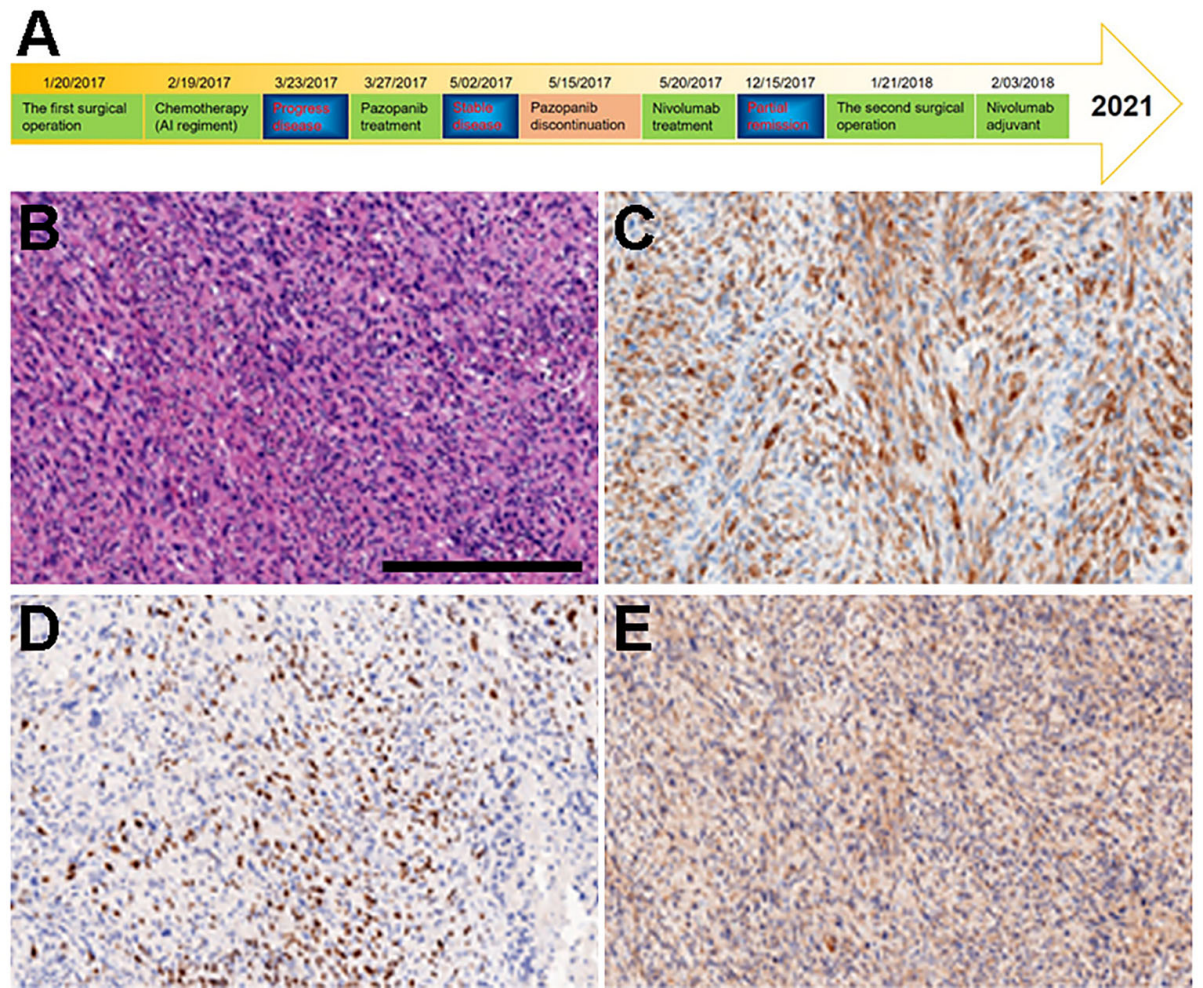
Diagnosis and treatment schematic plot and IHC of primary tumor tissue obtained from surgery. **(A)** Diagnosis and treatment schematic plot. **(B)** H&E. **(C)** DESMIN. **(D)** MYOD1. **(E)** MYGLB.

Here, we present a case of a patient with advanced PRMS who failed to respond to chemotherapy and antiangiogenic therapy but achieved a partial response (PR) with nivolumab treatment. Subsequently, the patient underwent R0 resection on the recurrent lesions, followed by nivolumab treatment as adjuvant and maintenance immunotherapy. At the time of submission of this manuscript, the patients achieved recurrence-free survival (RFS) lasting 45 months and counting (**Figure 1A**).

## Case Presentation

**Figure 2:**
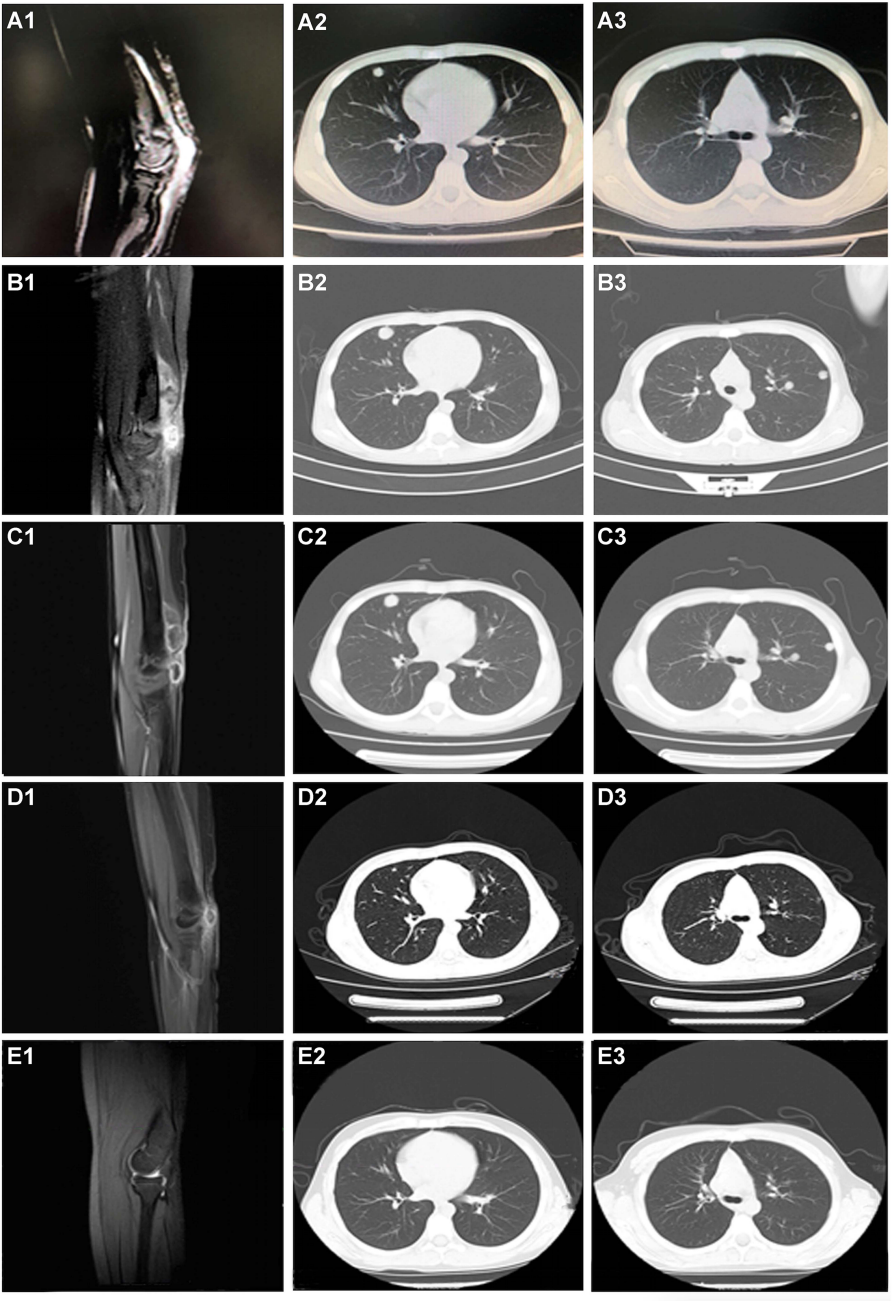
CT scanning during the whole treatment. **(A1–A3)** Before any systemic treatment. **(B1–B3)** After one cycle of chemotherapy and endostar. **(C1–C3)** After 1 month of pazopanib. **(D1–D3)** After four cycles of nivolumab. **(E1–E3)** The last followed up scanning. The first column was the limb viewport; the middle and last column was the lung viewport.

A 12-year-old Chinese male adolescent was referred due to a mass in his left elbow. In January 2017, surgical treatment was performed due to the rapid growth of the mass. Immunohistochemistry (IHC) results of the tumor specimens showed highly positive expression of DESMIN, MYOD1, and MYOGLB (**Figures 1B–E**) . *PAX3*-*FOXO* gene fusion was not detected by using fluorescence *in situ* hybridization (FISH). In February 2017, computed tomography (CT) of the chest and an enhanced CT scan of the left elbow revealed recurrence, showing two locoregionally relapsed lesions and multiple lung metastases (**Figures 2A1–A3**). Accordingly, the patient was diagnosed with poorly differentiated PRMS, stage IV (pT2N0M1).

Given his good physical condition, the patient was treated with endosarc (a recombinant human endostatin) in combination with chemotherapy (doxorubicin and ifosfamide). Progressive disease (PD) and serious treatment-related adverse events, such as nausea and myelosuppression, occurred after one cycle of chemotherapy (**Figures 2B1–B3**). This indicated a failure of the first-line chemotherapy. Starting on March 27, 2017, pazopanib monotherapy was administered following a multidisciplinary discussion. Although stable disease (SD) was achieved 1 month later (**Figures 2C1–C3**), the patient experienced severe adverse effects, including asthenia, myelosuppression, and nausea. The use of pazopanib was discontinued.

**Figure 3:**
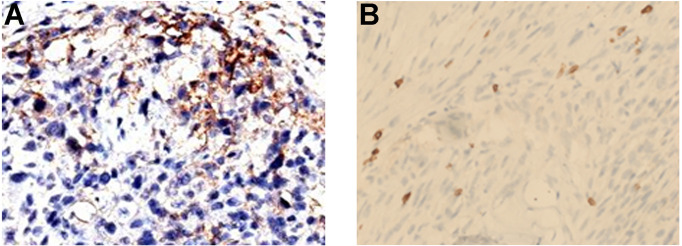
IHC assay of tumor tissue obtained from the first surgery. **(A)** PD-L1. **(B)** CD8.

Under the consent of the patient’s guardian, the surgical tissue sample was tested for the feasibility of immunotherapy. More than 30% of the tumor cells expressed PD-L1 (as tested by the SP263 assay on the Ventana platform), and tumor-infiltrating T cells appeared in the tumor region (**Figure 3**). Whole exon sequencing (WES) revealed a microsatellite-stable (MSS) status and HLA-I locus heterozygosity. The tumor mutation burden (TMB) was 2.05 mut/Mb (**Table 1**). Based on these findings, nivolumab monotherapy (100 mg Q2W) was initiated on May 20, 2017. CT scans exhibited a significant remission of the lung lesions after four courses of nivolumab treatment. One of the primary lesions had PR, but the other had little change (**Figures 2D1–D3**). Taken together, the total tumor volume was reduced by 87%, demonstrating that PR was achieved.

**Table 1:** The results of WES.

	Primary surgical tissue	Second surgical tissue
Somatic variations	TP53 p.R213*	TP53 p.R213*
	*SMAD2 p.P305A*	*SMAD2 p.P305A*
		*JUN p.A111-F114del*
Tumor mutation burden	2.05 Muts/Mb	4.79 Muts/Mb
Microsatellite instability	MSS	MSS
Neogenic antigen	32	54

*Nonsense mutation.

On January 21, 2018, the patient underwent R0 resection after 19 cycles of nivolumab immunotherapy. Another WES was performed on the surgical tumor tissue obtained during the second surgery and showed a TMB of 4.79 mut/Mb (**Table 1**). Upon submission of this manuscript, the patient continued to receive maintenance monotherapy with nivolumab. The most recent CT scan indicated no recurrence or metastasis. The patient has achieved an RFS of 45 months and counting (**Figures 2E1–E3**). Moreover, the toxicity associated with nivolumab treatment was tolerable through the whole course of immunotherapy.

## Discussion

Unlike other RMS subtypes, PRMS is a rare subtype that is noted more commonly in adults rather than in children and teenagers (1). There are no effective treatments and accurate prognostic biomarkers for PRMS, especially for refractory disease. Here, we reviewed the clinical courses and prognosis of 15 patients with metastatic or recurrent PRMS (**Table 2**) and found that surgical resection was still the best option. Adjuvant therapy, such as chemotherapy and immunotherapy, would benefit the patients further. The majority of patients died within a year of diagnosis. Nonetheless, long-term event-free survival has been achieved in two patients with adjuvant immunotherapy following surgical resection or immunotherapy alone (15). These two patients, in particular, were younger than the others. This indicates that ICI-based immunotherapy could be a promising treatment option for refractory PRMS, especially in teenagers. According to WHO classification, PRMS was not classified as a separate disease diagnosis in pediatric patients but was categorized as incorporated in ERMS diffuse anaplasia (23, 24). This highlights that age is a vital risk stratification factor for RMS.

**Table 2:** Fifteen cases of refractory PRMS including this one.

No.	Age	Location	Treatment	Metastasis and recurrent	Prognosis	Reference
1	80	Neck	Surg, chemo	Skin	Died of disease	(13)
2	70	Left thigh	Chemo	Lung	Alive without disease 12 months	(14)
3	19	Right thigh	Immu	Lung	Durable CR after 12 months	(15)
4	78	Sacrum	Surg	Liver, lung	Died of disease after 12 months	(16)
5	43	Leg	NA	Lung	Died of disease after 6 months	(17)
6	77	Arm	Surg, chemo	Regional LN	Alive without disease 13 months	(18)
7	72	Scalp	Surg, chemo	Skin, regional LN	Alive uncertain disease status 23 months	(18)
8	78	Leg	Surg	Lung	Died of disease after 12 months	(18)
9	41	Orbit	Surg, chemo	Lung, bone, local recurrence	Died of disease after 6 months	(19)
10	65	Submandibular	Surg, chemo	Local recurrence	Alive without disease 12 months	(20)
11	89	cheek	Surg	Local recurrence	Died of disease after 2 months	(21)
12	90	Uterine	Surg	NA	Dies of disease after 6 months	(14)
13	80	Uterine	Surg, chemo	NA	Died of disease after 6 months	(14)
14	50	Back	Surg, chemo	Regional LN	Alive with disease 2 months	(22)
15	12	Left elbow	Surg, immu	Lung metastasis	Durable CR after 45 months	Present case

Chemo, chemotherapy; Immu, immunotherapy; LN, lymph nodes; NA, not available; Surg, surgery.

The expression of PD-L1 in tumor cells correlates with inferior prognosis in STS patients, suggesting that the PD-1/PD-L1 axis could be a promising therapeutic biomarker (25). In many advanced malignant tumors, including STS (11, 26), monoclonal antibodies targeting the PD-1/PD-L1 axis have demonstrated considerable antitumor effects with a controllable toxicity profile. One of the essential approaches to improve the therapeutic benefit is to stratify patients using reliable biomarkers (27, 28).

In many solid tumors, such as non-small-cell lung cancer (NSCLC), PD-L1 expression in tumor cells and tumor-infiltrating lymphocytes (TILs) have strong predictive effects for anti-PD-1/L1 therapy (8). Little is known about the predictive significance of PD-L1 expression in STS. In a phase 2 study of pembrolizumab in advanced STS and bone sarcoma, three patients (4%) were identified as PD-L1 positive (TPS >1%) (9). Two of the three PD-L1-positive patients achieved an evaluable response: one complete response (CR) and one PR. Moreover, in another phase 2 clinical trial evaluating the efficacy and safety of cyclophosphamide plus pembrolizumab in 57 STS patients (29), only one patient presented a high level of PD-L1 expression (immune proportion score >10%) and achieved PR. A favorable response was also observed in a 17-year-old PD-L1-positive patient (TPS = 75%) with metastatic histiocytic sarcoma treated with nivolumab (30). Moreover, Tlemsani et al. reported the first case of a patient with PRMS (TPS = 60%), high levels of PD-L1 expression, and mismatch repair deficiency (dMMR). This patient was administered nivolumab monotherapy and achieved a long-term CR (15). Given the above data, PD-L1 expression may serve as a predictive biomarker for anti-PD-1/L1 treatment in STS patients.

The PD-L1 expression in STS patients varied among the studies. According to Perisano et al., 68.3% of high-grade sarcomas displayed positive PD-L1 expression (31). Chowdhury’s study revealed that RMS had the highest (86%) PD-L1-positive expression (TPS > 5%) when compared to other common pediatric tumors (32). Similar to the clinical result in NSCLC (33), our study also found that the proportion of CD8^+^ TIL seemed to be positively correlated with PD-L1 expression, implying that CD8^+^ TIL infiltration could become another effective indicator of immune response (32). The patient in our case presented with positive PD-L1 expression (TPS = 30%) and increased infiltration of CD8^+^ T cell (**Figure 3**). Given the impressive clinical response, we hypothesized that PD-L1-positive expression and/or TILs could be useful biomarkers for predicting the response to PD-1 blockade in PRMS.

Deficient mismatch repair/microsatellite instability-high (dMMR/MSI-H) has been identified as a powerful predictor of ICIs in multiple solid tumors, particularly colorectal and gastric cancer (8–11). The prevalence of dMMR/MSI-H in STS is 2.3% (34). In Doyle’s study, no response was observed in three dMMR PRMS patients treated with pembrolizumab. Lewin et al. reported that two dMMR cases with alveolar soft part sarcoma responded to durvalumab (anti-PD-L1) alone or in combination with tremelimumab (anti-CTLA-4) (35). The patient in our study was microsatellite stable (MSS) but benefited from nivolumab. Thus, the predictive value of dMMR/MSI-H for STS with anti-PD-1/L1 treatment remains debatable. In Doyle’s study, dMMR patients exhibited significantly higher TMB than proficient mismatch repair (pMMR) patients, consistent with previous clinical studies (36). High TMB has been reported as a potential biomarker to screen patients who may benefit from ICI therapy (37). However, TMB has no predictive ability for STS with anti-PD-1/L1 treatment due to the small patient population (35). Studies with larger sample sizes are needed to assess the predictive effects of dMMR/MSI-H and TMB in STS and PRMS.

Moreover, prior pazopanib treatment may have reinforced the brilliant response to nivolumab. The combination of atezolizumab (PD-L1 antibody) and bevacizumab [vascular endothelial growth factor (VEGF) antibody] has been recommended as the preferred first-line systemic therapy regimen in hepatocellular carcinoma (HCC) based on the favorable objective response rate (ORR) and survival benefit reported (38). Similarly, the clinical study of lenvatinib plus pembrolizumab demonstrated an impressive ORR. By blocking the FGFR4-GSK3β axis, lenvatinib reshaped Treg differentiation and reduced tumor PD-L1 levels, resulting in improved anti-PD-1 efficacy (39). In this case, the patient achieved SD after receiving pazopanib treatment (**Figures 2C1–C3**). Although the TKI was terminated due to severe adverse effects 1 month later, it is rational to speculate that pazopanib may have contributed to refining the tumor immune microenvironment and paved the way for the effects of nivolumab. Our findings suggest that TKI plus immunotherapy might be an effective option for PRMS.

## Conclusion

Here, we described a Chinese male adolescent PRMS patient with positive PD-L1 expression and TILs who achieved a remarkable response to nivolumab following TKI therapy. The PD-L1 and CD8 statuses were informative in guiding therapy decisions for this patient. Our findings could pave the way for a better understanding of the predictive biomarkers of anti-PD-1 therapies in PRMS.

## Data Availability Statement

The original contributions presented in the study are included in the article/supplementary material. Further inquiries can be directed to the corresponding author.

## Ethics Statement

The studies involving human participants were reviewed and approved by Peking University Cancer Hospital. Written informed consent to participate in this study was provided by the participants’ legal guardian/next of kin.

## Author Contributions

JL conceived the study. PL Liu took care of the patient. YL supervised patient care. FG, YT, and XZ from 3DMed Clinical Laboratory Inc. performed WES and immunohistochemistry on tumor tissue. JL evaluated and prepared images for the manuscript. All authors contributed to the article and approved the submitted version.

## Funding

This work was supported by grants from the National Natural Science Foundation of China (Grant No. 81802689).

## Conflict of Interest

Authors FG, YT and XZ are employed by 3DMed Clinical Laboratory Inc.

The remaining authors declare that the research was conducted in the absence of any commercial or financial relationships that could be construed as a potential conflict of interest.

## Publisher’s Note

All claims expressed in this article are solely those of the authors and do not necessarily represent those of their affiliated organizations, or those of the publisher, the editors and the reviewers. Any product that may be evaluated in this article, or claim that may be made by its manufacturer, is not guaranteed or endorsed by the publisher.

## Abbreviations

CT, computed tomography; dMMR, mismatch repair deficiency; ICI, immune checkpoint inhibitor; IHC, immunohistochemistry; MSI-H, microsatellite instability-high; MSS, microsatellite-stable status; MTI, medication time interval; PD, progressive disease; PD-1, programmed death protein 1; PR, partial response; PRMS, pleomorphic rhabdomyosarcoma; RMS, rhabdomyosarcoma; SD, stable disease; STS, soft tissue sarcoma; TIL, tumor-infiltrating lymphocytes; TMB, tumor mutation burden; WES, whole exon sequencing.
